# Recent advances in celiac disease and refractory celiac disease

**DOI:** 10.12688/f1000research.18701.1

**Published:** 2019-06-26

**Authors:** Georgia Malamut, Sascha Cording, Nadine Cerf-Bensussan

**Affiliations:** 1 Gastroenterology, Hôpital Cochin APHP, Paris, France; 2 Université Paris Descartes, Paris, France; 3 Inserm, UMR1163 and Institut Imagine, Laboratory Intestinal Immunity, Paris, France

**Keywords:** celiac disease, refractory celiac disease

## Abstract

Celiac disease (CeD), defined as gluten-induced enteropathy, is a frequent and largely underdiagnosed disease. Diagnosis relies on the detection of highly specific serum IgA anti-transglutaminase auto-antibodies and on the demonstration of duodenal villous atrophy. Treatment necessitates a strict gluten-free diet, which resolves symptoms and enables histological recovery. However, regular follow-up is necessary to assess mucosal healing, which emerges as an important prognostic factor. Recent work on CeD pathogenesis has highlighted how the cross-talk between gluten-specific CD4 ^+^ T cells and interleukin-15 can activate cytotoxic intraepithelial lymphocytes and trigger epithelial lesions. Moreover, acquisition by a subset of intraepithelial lymphocytes of somatic gain-of-function mutations in the JAK-STAT pathway was shown to be a decisive step in the progression toward lymphomas complicating CeD, thus opening new therapeutic perspectives for these rare but life-threatening complications.

## Introduction

Celiac disease (CeD) is defined as a chronic immune-mediated small intestinal enteropathy driven by dietary gluten in genetically predisposed individuals carrying HLA-DQ2 or HLA-DQ8. Serological testing has revealed its broad clinical spectrum, worldwide distribution, and increasing incidence in industrialized countries ^1^. In most patients, a gluten-free diet (GFD) allows mucosal repair in one or two years ^2^. However, a relationship has recently been established between lack of mucosal healing and risk of complications ^3, 4^. Precise diagnosis of the cause of persistent atrophy is crucial for guiding therapy. Poor adherence to GFD, which is observed in 50% of patients ^5^, and non-celiac causes of enteropathy must be eliminated. Refractory celiac disease (RCD), which complicates CeD in about 1% of cases, and overt lymphoma must be considered. These conditions and their pathogenesis are discussed below. CeD has emerged as a model disease to dissect how chronic activation of the gut immune system by dietary antigens can progressively overcome the potent mechanisms of tolerance, which maintain gut homeostasis, lead to tissue damage, and ultimately promote lymphomagenesis.

## Clinical presentation and diagnosis

The classic presentation of CeD with diarrhea, abdominal pain, and severe malnutrition has become rare, and many patients now manifest with non-digestive symptoms, notably anemia, osteoporosis, or arthralgia (reviewed in 6). CeD is also now frequently revealed by serological screening of at-risk individuals, including first-degree relatives and patients with autoimmune diseases, notably type I diabetes or autoimmune thyroiditis (reviewed in 6). Depending on duodenal histology and symptoms severity, CeD is defined as potential, asymptomatic, symptomatic, classic, non-classic, or refractory (reviewed in 6).

The first diagnostic step consists of the detection of serum anti-transglutaminase-2 (TG2) IgA (or anti-TG2 IgG in case of IgA deficiency that is present in up to 3% patients with CeD). This serological test is highly specific, sensitive, and less expensive than dosage of serum anti-endomysial antibodies (reviewed in 6). When serology is positive, duodenal biopsies are needed to confirm diagnosis. Bulb biopsy is now recommended in addition to duodenal biopsy because of evidence of ultra-short CeD ^7^. In children, new criteria for diagnosis have been defined by the European Society of Pediatric Gastroenterology, Hepatology, and Nutrition. Accordingly, biopsy is no longer required if anti-TG2 concentrations are 10-fold the upper normal value in children, who are HLA-DQ2.5 or DQ8 and also have positive serology for anti-endomysial IgA ^8^. In adults, reference biopsies are advisable since villous atrophy can persist over a long period (6 months to 5 years, median of 1.3 years) in more than 40% of patients on GFD ^2^, and lack of mucosal healing has been associated with increased risk of complications, notably bone fractures and lymphomas ^3, 4^. The latter data lead to the recommendation of annual biopsy follow-up until complete villous recovery, even in asymptomatic patients. Persistent intestinal villous atrophy should prompt clinicians to check for adherence to GFD and, in case of strict compliance, to exclude RCD.

Two types of RCD have been described according to the normal (type I RCD = RCDI) or abnormal (type II RCD = RCDII) phenotypes of intraepithelial lymphocytes (IELs). In contrast to CeD, RCD is almost always symptomatic and diagnosis is suspected because of persistent symptoms and lack of histological recovery after GFD. Malnutrition can be particularly severe in RCDII due to ulcerative jejunitis. Thus, large ulcers are observed in 70% of patients and cause protein loss enteropathy with severe chronic diarrhea and hypoalbuminemia ^9^. Presentation of RCDI is less severe and usually mimics active CeD. Jejunal ulcers are absent or very small ^9^. By revealing large ulcers, capsule endoscopy can help to differentiate RCDI and RCDII but this exam is contraindicated in case of strictures ^10^. RCDI diagnosis requires negative celiac serology and confirmation of strict adherence to GFD by a dietitian ^9^. As of recently, novel urine and stool tests that enable quantitative detection of gliadin immunogenic peptides early after ingestion of gluten can be used to assess dietary compliance ^11, 12^. RCDII diagnosis is more codified since it is based on the demonstration of a clonal population of IELs with a distinctive phenotype (described below). Primary resistance to GFD is observed in 30% of RCDI and 50% of RCDII. In RCDII, retrospective demonstration of a switch from normal to abnormal clonal IELs is difficult and was made only twice in our cohort ^9^. Of note, some primary cases of RCDII may become asymptomatic and show partial villous recovery after GFD, pointing to the key role of gluten exposure in driving RCDII (personal observation and see below).

**Figure 1.**
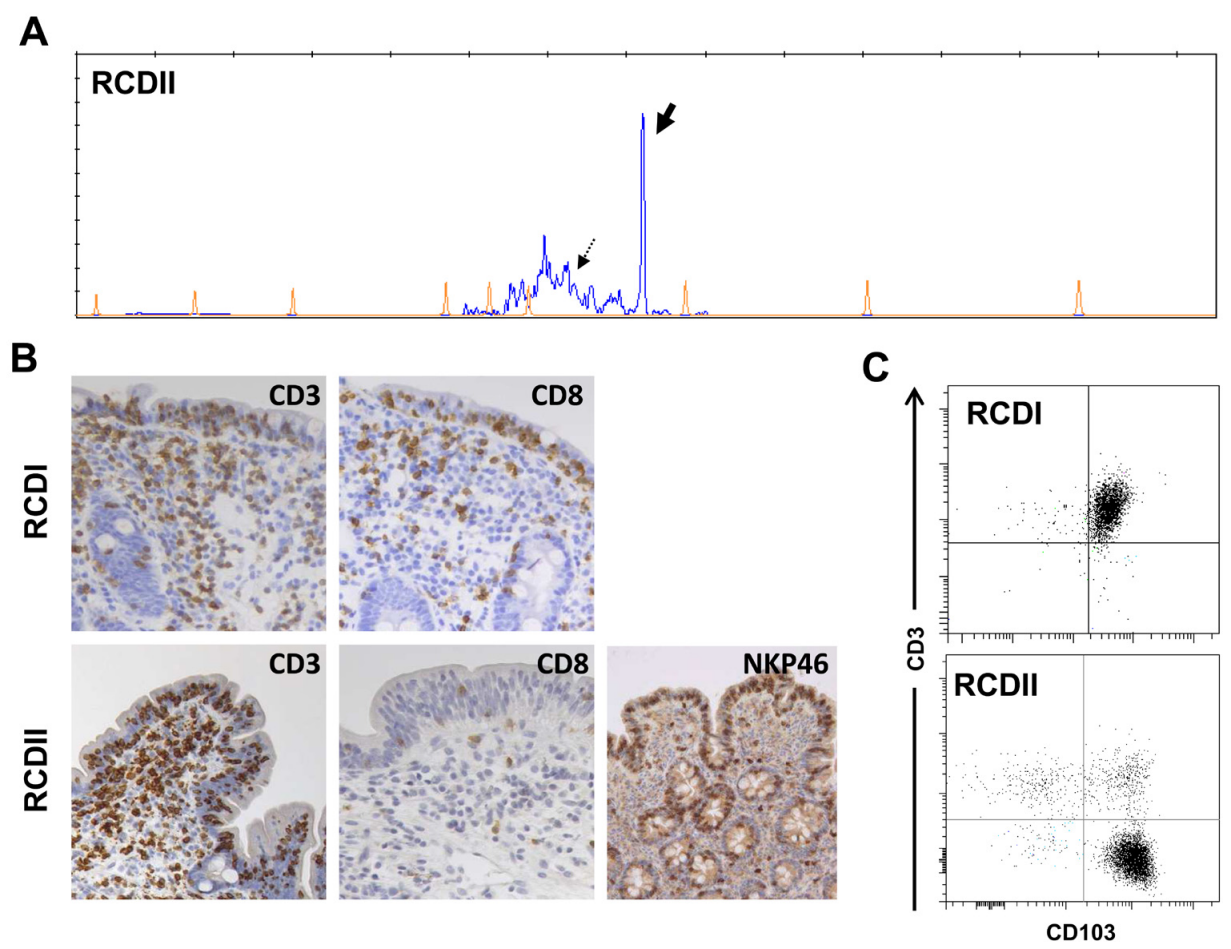
Multidisciplinary approach for differential diagnosis of type I and II refractory celiac disease. ( **A**) Detection by multiplex polymerase chain reaction of clonal T-cell receptor gamma (TCRγ) chain rearrangement (peak indicated by thick arrow) in duodenal biopsies of type II refractory celiac disease (RCDII). The polyclonal profile (thin arrow) corresponds to normal resident T cells. RCDI biopsies show only a polyclonal profile (not shown). ( **B**) Analysis of intraepithelial lymphocyte (IEL) phenotype by immunohistochemistry in paraffin sections. In RCDI, the majority of IELs are CD3 ^+^ and CD8 ^+^ (upper panel); in RCDII, IELs contain CD3 but generally lack CD8 (lower panel). NKP46 is a useful diagnostic marker as expressed by a majority of malignant IELs in RCDII (lower panel) but only by a minority of normal T-IELs in celiac disease and RCDI (not shown). ( **C**) Flow cytometry on IELs isolated from duodenal biopsies. When possible, it provides precise assessment of IELs phenotype and notably allows a clear distinction between normal CD103 ^+^ IELs with surface CD3 in RCDI (upper panel) and abnormal CD103 ^+^ IEL lacking surface CD3 in RCDII (lower panel). The immunochemistry photos were reused in this figure with permission.

Diagnosis of RCDII benefits from a multidisciplinary approach. Immunohistochemistry can differentiate abnormal IELs, which contain CD3 but generally lack CD8, from normal CD8 ^+^ T-IELs. Flow cytometry on IELs isolated from intestinal biopsies is useful, especially if the frequency of abnormal IELs is less than 50%, in order to demonstrate the absence of surface CD3 and T-cell receptor (TCR) ^13^ and the frequent expression of the natural killer (NK) receptor NKP46 ^14^. Diagnosis is confirmed by demonstrating clonal TCR gamma or delta chain rearrangements by using multiplex polymerase chain reaction on DNA extracted from biopsies ( Figure 1) ^9, 15^. The possibility of using paraffin-embedded biopsies to demonstrate clonality and to detect abnormal NKP46 ^+^ IELs is a recent advance that should facilitate RCDII diagnosis in non-specialized centers ^15, 16^. As highlighted below, RCDII is a malignant condition now regarded as a low-grade intraepithelial lymphoproliferation. Intestinal and extra-intestinal diffusion of the malignant cells is frequent and can be monitored in blood and in different organs by testing for the clonal rearrangement and the presence of lymphocytes displaying the same abnormal phenotype and expressing CD103, a marker characteristic of IELs ^9, 13^ ( Figure 1).

Extensive work-up may be necessary to eliminate other enteropathies refractory to GFD. RCDI should be distinguished from olmesartan-induced enteropathy, which necessitates the avoidance of this anti-hypertensive drug ^17^, but also from autoimmune enteropathy, which requires a search for an underlying primary immunodeficiency that may benefit from specific therapy ^18– 20^. RCDII must be differentiated from rare cases of CeD complicated by large lymphocytic leukemia infiltrating the intestine ^21^ but also from intestinal small T-cell lymphomas ^22^, which similarly can present with severe chronic diarrhea, low albuminemia, intestinal villous atrophy, and clonal TCR rearrangement. In the latter cases, flow cytometry can be helpful to identify the phenotype of malignant cells and reach diagnosis ^21, 22^.

## Epidemiology of celiac disease and refractory celiac disease

About 1% of the worldwide population is affected by CeD, but there are differences between and within countries. In the US, CeD is more frequent among non-Hispanic whites than among non-Hispanic blacks and Hispanics ^1, 23^. Prevalence in Asian countries is overall comparable to that in Europe but varies largely depending on the frequency of HLA-DQ2 and -DQ8 and wheat intake. Accordingly, the prevalence of CeD is high in Western Asia and Northern India but lower in South India and South and South Eastern Asia, where rice is the staple food and HLA-DQ2 is less frequent ^24, 25^.

RCD incidence remains unknown. A North American referral center suggests a cumulative incidence of 1.5% for both RCDI and RCDII among patients with CeD ^26^. In the Derby cohort, West ^27^ reported 0.7% RCD cases with ulcerative jejunitis among 713 patients with CeD. The respective proportion of RCD subtypes is also ill defined and may differ between countries, RCDI being more frequently diagnosed in North America ^28^ and RCDII in European countries ^29– 31^. It is unclear whether differences can be explained by a distinct prevalence of HLA-DQ genotypes, HLA-DQ8 being more frequently associated with CeD in New York than in Paris ^32^, or a higher gluten consumption in Southern than in Northern Europe (discussed in 33).

## Pathophysiology of celiac disease and refractory celiac disease

### The anti-gluten CD4 ^+^ T-cell response

**Figure 2.**
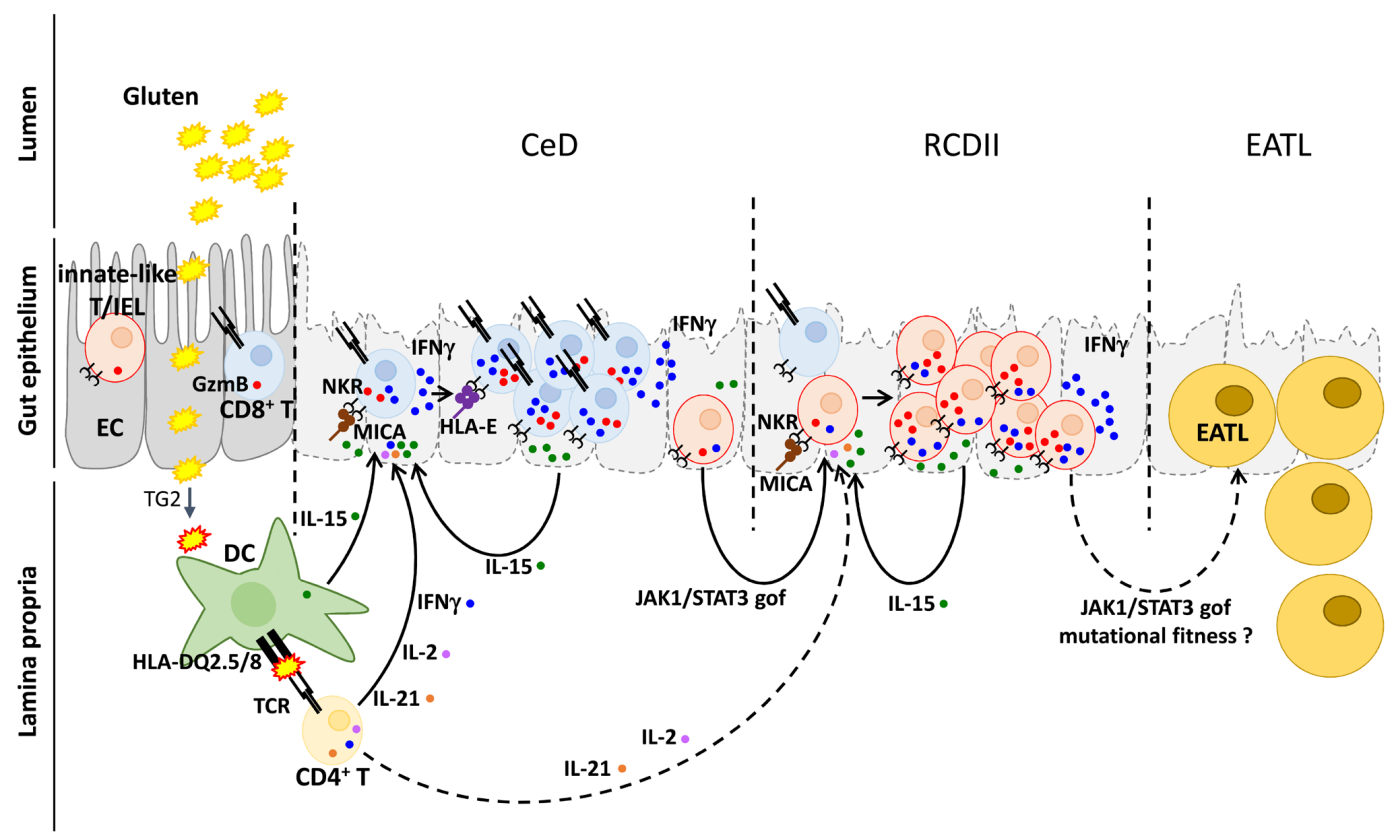
Mechanisms driving activation and malignant transformation of intraepithelial lymphocytes in celiac disease and type II refractory celiac disease. In celiac disease (CeD) and type II refractory CeD (RCDII), CD4 ^+^ T cells are activated by gluten peptides modified by transglutaminase-2 (TG2) and loaded onto HLA-DQ2.5/DQ8 molecules at the surface of antigen-presenting cells. Activation of CD4 ^+^ T cells harboring cognate T-cell receptors (TCRs) for gluten peptides is likely initiated by dendritic cells (DCs) in gut-lymphoid tissue or mesenteric lymph nodes (not shown). Primed gluten-specific CD4 ^+^ T cells may then home into the gut *lamina propria*. Upon reactivation by gluten peptides presented by DCs or perhaps by plasma cells ^36^, the latter cells secrete cytokines—interleukin-2 (IL-2), IL-21, and interferon gamma (IFNγ)—which can cooperate with IL-15, produced notably by epithelial cells (ECs), to activate cytotoxic intraepithelial lymphocytes (IELs) and license enterocyte killing. In uncomplicated CeD, IL-2 and IL-21 cooperate with IL-15 to stimulate cytotoxic CD8 ^+^TCRαβ ^+^ IELs expressing natural killer receptors (NKRs). In RCDII, somatic *JAK1* or *STAT3* gain-of-function (gof) mutations, which confer hyper-responsiveness to IL-15, IL-2, and IL-21, allow a clone of innate-like T-IELs to progressively out-compete normal T-IELs and invade the epithelium. Major histocompatibility complex class I polypeptide-related sequence A (MICA), which is induced by stress, and HLA-E which is induced by IFNγ, are two NKR ligands that are upregulated on ECs in active CeD and in RCD. Their expression promotes enterocyte killing by T-IELs in CeD, and by malignant innate-like T-IELs in RCDII. During their expansion in the gut epithelium, RCDII IELs can acquire additional mutations, which promote their transformation into aggressive enteropathy-associated lymphoma (EATL).

Past studies have clearly demonstrated the central role of anti-gluten CD4 ^+^ T-cell immunity in CeD pathogenesis, thereby establishing the link between the main environmental trigger, dietary gluten, and the main genetic risk factor, the major histocompatibility complex (MHC) class II molecules. Thus, 90% of the patients express HLA-DQ2.5 (DQA1*05-DQB1*02) and most of the remaining patients express HLA-DQ8 (DQA1*03-DQB1*03:02) or HLA-DQ2.2 (DQA1*02:01-DQB1*02) (reviewed in 34, 35) ( Figure 2).

Gluten is the viscoelastic blend obtained by mixing flours from wheat, barley, or rye with water, which is used to make bread and pasta. It comprises hundreds of proteins displaying repeated sequences rich in proline and glutamine ^37^. These proteins are incompletely digested in the gut lumen and release peptides, which can reach the subepithelial tissue and bind HLA-DQ2.5/8 molecules at the surface of intestinal antigen-presenting cells. As a consequence, gluten peptides are presented to and activate specific CD4 ^+^ T cells (reviewed in 34, 35) ( Figure 2). Most patients with CeD respond to a limited and shared set of peptides thereby defined as public or immunodominant epitopes ^38, 39^. A larger number of these epitopes are recognized in the context of HLA-DQ2.5 than in that of HLA-DQ8, likely accounting for the preferential association of CeD with HLA-DQ2.5. Immune recognition of the gluten epitopes is highly dependent on their post-translational modification by TG2, which converts neutral glutamine into negatively charged glutamic acid residues within the intestinal mucosa. This modification enhances gluten epitope avidity for HLA-DQ2/8, thus allowing the formation of stable HLA-DQ–gluten complexes that are critical for efficient T-cell engagement and activation (reviewed in 34, 40).

Recent technical developments have enabled further characterization of the T- and B-cell immune responses elicited by gluten. A very high proportion of the gut plasma cells, which expand massively in the *lamina propria* during active CeD, were shown to produce IgA specific for gluten or TG2 or both. HLA-DQ molecules complexed gluten T cell epitopes as well as co-stimulatory molecules were detected at their surface, leading to suggest their role in gluten presentation to T cells ^36, 41, 42^. Observation of IgA ^+^ DQ2.5-glia-α1a presenting cells among TG2-specific plasma cells also strengthens the so-called hapten-carrier hypothesis as a mechanism by which TG2 specific B cells get help from gluten reactive T cells. This hypothesis was proposed by Sollid to explain why anti-TG2 auto-antibodies provide a serum signature specific for active CeD and disappear after GFD ^40^. Gluten-specific CD4 ^+^ T cells have been extensively characterized. They produce large amounts of cytokines, notably interferon gamma (IFNγ) and IL-21 (reviewed in 34, 35). They possess a polyclonal TCR repertoire but preferentially use some variable (V)-gene segments and frequently display a non-germline, positively charged arginine residue in the highly variable CDR3 region of the Vβ chain ^38, 39, 43^. The biased use of TCR-Vα chain segments is thought to reflect their preferential interaction with HLA-DQ. The arginine in the CDR3β loop might act as a lynchpin in the peptide–HLA-DQ interaction (discussed in 38). Fluorescent tetramers made of HLA-DQ2.5 molecules bound to immunodominant gluten epitopes have been designed to track gluten-specific CD4 ^+^ T cells *in vivo* ^44^, and single-cell sequencing was used to follow-up changes in their repertoire during the patients’ life ^45^. This elegant combination of approaches showed that gluten-specific CD4 ^+^ T cells clonally expand in the gut of untreated patients, where they represent about 2% of CD4 ^+^ T cells. A very small number of the latter cells circulate in blood, where they decrease after GFD. Yet memory gluten-specific CD4 ^+^ T cells persist in blood for decades despite GFD and can re-expand rapidly after oral gluten challenge, explaining the need for definitive GFD to prevent relapse ^45^. Overall, the gluten-specific T-cell response is now well characterized and its role in CeD pathogenesis undisputable. Therefore, many efforts are made for developing alternative treatments to GFD which may prevent this response, notably using oral enzymes ^46^, TG2 blockers ( https://zedira.com), or peptide-based immunotherapy to desensitize patients ^47^.

### The role of cytototoxic intraepithelial lymphocytes and interleukin-15 in tissue damage

If gluten-specific CD4 ^+^ T cells are indispensable to trigger CeD, it is now clear that tissue damage also requires the activation of cytotoxic IELs in the presence of interleukin-15 (IL-15). A massive expansion of IELs and notably of CD8 ^+^ IELs with an αβ TCR is one of the cardinal features of active CeD ( Figure 2). IL-15 has emerged as a key player in CeD. This cytokine, notably produced by intestinal epithelial and dendritic cells, stimulates IEL expansion and cytotoxic activity and impairs their negative regulation by regulatory T cells and transforming growth factor beta (TGF-β) (reviewed in 34, 35). Studies in mouse models further indicate that upregulation of IL-15 and activation of intestinal CD4 ^+^ T cells by the dietary antigen are both necessary to drive the cytotoxic activation of CD8 ^+^TcRαβ ^+^ IELs and epithelial damage (reviewed in 48). The mechanism of cooperation is not yet definitively demonstrated but was ascribed in one mouse model to the synergistic effects of IL-15 and of IL-2 produced by antigen-specific CD4 ^+^ T cells ^49^. In this model, the two cytokines were sufficient to drive the expansion of IELs and to enhance their expression of granzyme B and of NK receptors independently of any direct antigen recognition. These results are reminiscent of findings in CeD. Thus, in active CeD, CD8 ^+^ T-IELs display enhanced expression of granzyme B and of the activating NK receptors NKG2D and CD94-NKG2C. In the presence of IL-15 *in vitro*, these cells can kill targets expressing MICA and HLA-E, the respective ligands of these receptors (reviewed in 34, 35). This scenario may operate during active CeD, when expression of MICA, HLA-E, and IL-15 is upregulated in the duodenal epithelium ^50, 51^. In CeD, it is likely that not only IL-2 but also IL-21 that is produced by gluten-specific CD4 ^+^ T cells can cooperate with IL-15 to drive IEL activation ( Figure 2).

A second subset of T-IELs characterized by the expression of a γδ TCR has attracted much attention in CeD. Indeed, TCRγδ ^+^ IELs expand in patients with potential CeD before the development of epithelial lesions and their number remains increased long after initiating GFD (reviewed in 35). Recent work indicates that the repertoire of resident TCRγδ ^+^ cells in various tissues is shaped by butyrophilin-like molecules (BTNLs) expressed by epithelial cells ^52^. In the human gut, expression of BTNL3 and BTNL8 drives the selective expansion of resident Vγ4 ^+^/Vδ1 ^+^ IELs ^52^. Strikingly, in active CeD, the gut epithelium loses BTNL8 expression and this loss is associated with a profound depletion of resident Vγ4 ^+^/Vδ1 ^+^ IELs that are replaced by a distinct subset of Vδ1 ^+^ IELs. The latter cells failed to recognize BTNL3/BTNL8 and displayed a distinct functional program dominated by the production of IFNγ. Avoidance of dietary gluten restored BTNL8 expression but was insufficient to reconstitute the physiological resident Vγ4 ^+^/Vδ1 ^+^ IELs subset among TCRγδ ^+^ IELs ^53^. Overall, these data show that chronic inflammation permanently reconfigures the tissue-resident TCRγδ ^+^ IELs compartment in CeD. However, the exact role of the latter cells in CeD pathogenesis remains elusive.

### Ongoing questions in celiac disease pathogenesis

Several questions remain to be answered. It is still unclear why only 5 to 10% of patients carrying predisposing HLA will develop gluten-specific CD4 ^+^ T cells and tissue damage. Besides HLA-DQ2.5 homozygosity, which increases the risk of CeD (reviewed in 40), about 50 CeD-associated common genetic variants, most of which are shared with other autoimmune diseases, have been identified by genome-wide studies. Yet their influence is modest overall and they bear no predictive value ^54, 55^. A possible role of the gut microbiota remains elusive. Yet changes in duodenal and fecal microbiota in active CeD and observations in mouse models suggest its influence on gluten-induced pathology, possibly via modulation of gluten degradation ^56, 57^. The long-lasting hypothesis of a viral trigger was recently examined in humanized mice carrying HLA-DQ8, which develop gluten-specific CD4 ^+^ T cells ^58^. Intestinal infection by a reovirus that induced type I interferon resulted in loss of systemic tolerance to gluten, although the mice did not develop any intestinal lesions ^58^. Recent epidemiological data from the TEDDY cohort of children at risk of CeD support the predisposing role of infections by gut-tropic viruses. Thus, risk of seroconversion increased within the three months following an episode of gastrointestinal infection whereas risk of CeD decreased in children vaccinated against rotavirus before three months of age ^59^. Finally, one unanswered question concerns the mechanism(s) of IL-15 upregulation in the intestine of patients with CeD. The inducing role of epithelial stress has been suggested ^60^ perhaps triggered by microbiota-derived innate signals or by peptides present in wheat or gluten ^27, 61^. Despite these interrogations, the mechanisms described above provide a plausible scenario and explain the efficacy of a strict GFD in the vast majority of patients with CeD. Yet primary or secondary resistance to GFD can develop in a small fraction of patients with RCD.

### Mechanisms of resistance to gluten-free diet in type I and type II refractory celiac disease

As indicated above, differential diagnosis of RCDI and RCDII is based on the presence or absence of a clonal population of IELs with an unusual phenotype. Accordingly, the intestine of patients with RCDI contains polyclonal T cells and immunophenotyping does not reveal any significant difference with uncomplicated CeD except for a moderate and inconstant increase in the percentage of CD4 ^+^ IELs ( 13 and personal data). Extra-intestinal autoimmunity is more frequent than in uncomplicated CeD, and disease is improved by immunosuppressive therapies. Therefore, we have suggested that autoimmune cells develop in the intestine of patients with RCDI and drive the persistence or relapse of intestinal lesions. However, this mechanism remains hypothetical. Long-lasting inflammation may promote the frequent development of collagenous sprue and predispose patients to the onset of overt lymphomas. Yet this severe complication is much less frequent than in RDII (reviewed in 13).

In contrast to RCDI, RCDII is now a well-characterized entity that can be defined as a low-grade clonal intraepithelial lymphoproliferation with a high risk of transformation into overt enteropathy-associated T-cell lymphoma (EATL) (reviewed in 13). In keeping with their intraepithelial origin, malignant IELs express the αE integrin (CD103), the expression of which may disappear with disease progression, notably in EATL (reviewed in 13 and personal observation). As indicated above, in most patients, malignant IELs lack expression of surface CD3-TCR complexes and CD8 but they contain intracellular CD3 and display clonal rearrangement of TCR genes ^13^. Conversely, the abnormal IELs express NK receptors, notably NKP46, and, in the presence of IL-15, they can kill enterocyte lines *in vitro* ^14, 50, 62^, a property which may explain the severe ulcerative jejunitis often observed in patients with RCDII ^13^. These characteristics of the malignant IELs are instrumental for diagnosis. They have also raised many speculations on their cellular origin. We have recently shown, contrary to many expectations, that they do not derive from the transformation of T-IELs but from a small subset of unusual innate-like T-IELs present in the normal intestine. Like their malignant counterpart, the latter cells do not express surface CD3 but contain intracellular CD3 and DNA rearrangements of the TCR, and they also possess NK receptors and NK functions ^14^. This unusual phenotype is imprinted by a combination of IL-15 and NOTCH signals during their differentiation in the gut epithelium from bone marrow precursors. Innate-like T-IELs form only a very small fraction of IELs in the normal adult intestine as well as in uncomplicated CeD, where most IELs are T lymphocytes ^14^. If almost all RCDII cases arise from a transformed clone of innate-like T-IELs, there are some exceptions. Thus, in rare RCDII cases, the clonal intraepithelial lymphoproliferation develops from TCRγδ ^+^ IELs or even from TCRαβ ^+^ IELs expressing (or not) CD8 (unpublished observations). Immunohistochemical detection of NKP46 can help diagnosis as this NK marker is expressed by malignant IELs in most cases of RCDII but by only a minority of normal T-IELs in CeD and RCDI ^16^.

Why may a clone of innate-like T-IELs (RCDII IELs) progressively expand at the expense of the normal polyclonal T-IELs, which massively infiltrate the gut epithelium of patients with CeD? Following work showing that IL-15 provides signals to RCDII IELs which promote their expansion and cytotoxic activation (reviewed in 13, 35), we recently showed that RCDII IELs frequently contain somatic *JAK1* or *STAT3* gain-of-function mutations (or both), which confer hyper-responsiveness to IL-15 ^14^. These mutations may also promote response to other cytokines present in the intestine of patients with CeD, notably IL-2 and IL-21, which are produced by gluten-activated CD4 ^+^ T cells ^63^. Thus, *JAK1* and *STAT3* mutations may enable transformed innate-like T-IEL to outcompete normal resident T lymphocytes in the cytokine-rich environment of the intestine of patients with CeD. Our ongoing work further suggests that RCDII IELs can acquire additional mutations that may promote their dissemination in and beyond intestine and ultimately lead to their transformation into aggressive EATL ( 13, 21 and unpublished observations).

Overall, these data provide much better insight into the mechanisms that drive lymphomagenesis in CeD. They also suggest possible therapeutic strategies, such as IL-15 blockade as tested in a recent international clinical trial ^64^ or alternatively the use of JAK inhibitors. However, possible risks are to impair a putative anti-tumoral response or to promote the clonal escape of malignant cells carrying additional mutations conferring a growth advantage or both. More work is necessary to delineate benefits and risks of these therapies. Another question concerns factors, which predispose patients with CeD to develop RCDII and EATL. The higher frequency of HLA-DQ2.5 homozygosity in RCDII (about 65%) than in uncomplicated CeD (about 30%) ^13^ points to a key role of the adaptive anti-gluten T-cell response. Accordingly, RCDII and EATL develop preferentially in patients with poor adherence to the diet or undiagnosed until late in life ^13^. Moreover, strict GFD, even if insufficient to treat RCDII, is indispensable to control malignant cell expansion and to reduce the epithelial damage that is induced by malignant cells ^13^. A recent genome-wide analysis in two small cohorts of patients with RCDII of Dutch or French origin also points to a predisposing genetic locus in 7p14.3 ( *P* = 2.37 Å ~ 10 ^−8^, odds ratio = 2.36), which may control expression of FAM188B ^65^. The exact contribution of this variant to CeD-associated lymphomagenesis remains elusive but interestingly FAM188B was recently implicated in the regulation of P53, which plays an important role in tumor surveillance ^66^.

## Treatment and outcome

GFD remains the standard treatment of CeD and is currently the only treatment to reduce inflammation and allow villous recovery. In patients with CeD, GFD prevents the onset of autoimmune diseases and lymphoproliferative disorders ^67, 68^. Accordingly, celiac patients with mucosal inflammation are at higher risk of mortality (10.8 per 1000 person-years) than those without mucosal damage (1.7 per 1000 person-years) ^69^. Given the social burden of GFD, many efforts are currently made for developing alternative treatments. The efficacy of oral enzymotherapy has not yet been proven ^46^. TG2 blockade ( https://zedira.com) or peptide-based immunotherapy to desensitize patients ^47^ is currently under assessment.

**Figure 3.**
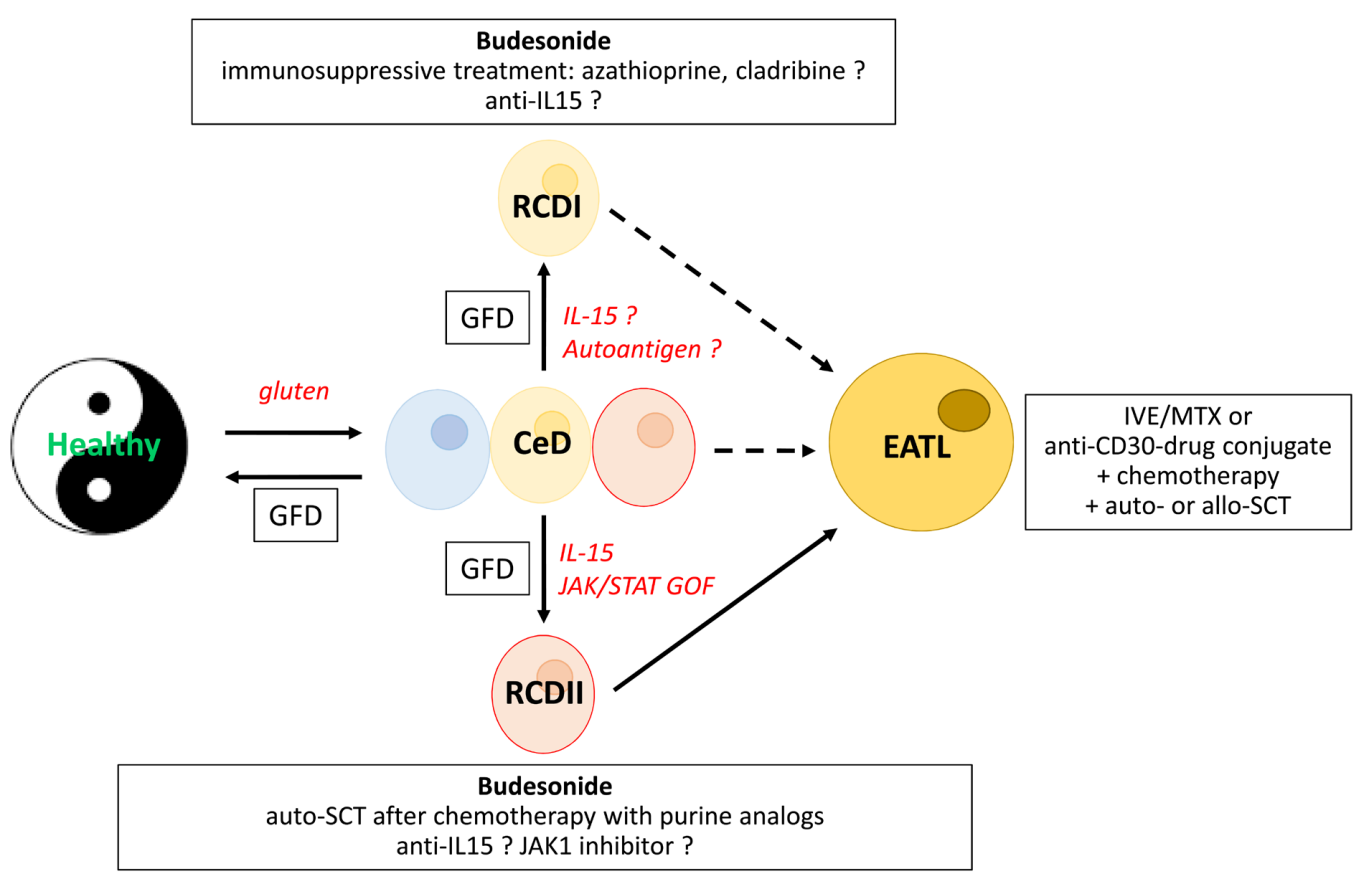
Therapeutic strategies in refractory celiac disease. Open-capsule budesonide is the first-line treatment in type I (RCDI) and type II (RCDII) refractory celiac disease. Immunosuppressive drugs can be used in steroid-dependent RCDI but not in RCDII. Autologous stem cell transplantation (auto-SCT) preceded (or not) by treatment with purine analogues can be proposed in RCDII before the age of 65 years. Targeted therapy with human anti–interleukin-15 (anti–IL-15) antibody is currently tested in RCDI and RCDII. JAK1 inhibitor may be considered in RCDII. At the stage of enteropathy-associated lymphoma (EATL), chemotherapy with anti-CD30 antibody coupled to a cytotoxic drug (brentuximab vedotin) followed by auto- or allogenic SCT (allo-SCT) is currently tested if malignant cells express CD30. For CD30-negative EATL, IVE/MTX (ifosfamide, vincristine, etoposide, and methotrexate) chemotherapy followed by auto-SCT can be used ^76^. CeD, celiac disease; GFD, gluten-free diet.

Strict GFD is also indispensable in RCD but complementary treatments are needed. In both RCDI and RCDII, the standard option consists of administration of open-capsule budesonide ^70^. This particular oral route allows spreading of budesonide in the proximal part of the small bowel, where mucosal damage is maximal. Budesonide allows clinical remission and villous recovery in around 90% of both types of RCD ^70^. In RCDI with steroid dependence, immunosuppressive drugs may be used. Owing to the risk of promoting overt lymphoma, they are no longer used in many centers for RCDII treatment ^9^. In RCDII, chemotherapy with purine analogs such as cladribine, pentostatine, or fludaribine can been used ^71, 72^, notably before autologous stem cell transplantation, which is a valuable therapeutic option in RCDII ^73^. Evidence of IL-15 contribution to RCDII pathogenesis has led to a recent clinical trial using human anti–IL-15 antibody ^64^. Demonstration of JAK1/STAT3 mutations ^14^ provides the rationale to test JAK inhibitors, but as discussed above, there are some caveats to both approaches ( Figure 3). The objectives of treating RCD are to cure malnutrition and to prevent onset of overt EATL. The risk of EATL is higher in RCDII (about 50% 5 years after diagnosis of RCDII) than in RCDI (less than 14%) ^9^. Patients with RCDII must be regularly followed up with upper/lower endoscopy with the optional usage of computed tomography scan or magnetic resonance image small bowel follow-through. Positron emission tomography is useful to detect EATL ^74^. Importantly, RCD is not a necessary step between CeD and EATL, as EATL can complicate known or unknown CeD and presents notably as a surgical emergency with small bowel obstruction or peritonitis ^75^. Whatever the underlying enteropathy, EATL is frequently multifocal and can present as a mesenteric mass. If EATL is localized, elective surgery may be useful to prevent complications during chemotherapy ^76^. For EATL expressing CD30 (80% of cases), chemotherapy is now combined with anti-CD30 antibody coupled to a cytotoxic agent ( Figure 3) ^77^. Small bowel carcinoma is another malignant complication but largely less frequent than lymphomas. Thus, a recent follow-up study of a cohort of 1138 patients with CeD during 25 years revealed 29 cases of RCD, and seven cases of EATL, but only four cases of small bowel carcinomas ^78^.

## Conclusions

CeD is frequent and has a mainly benign course under GFD. Lymphomatous complications are rare but their prognosis is poor because of a lack of efficient treatment. Absence of mucosal healing is an important risk factor for such complications. Recent advances in the pathophysiology of CeD and RCD open the possibility of targeted therapies but their efficiency and safety remain to be assessed.
